# Novel photochromic system using methylene blue reduction with l-ascorbic acid†

**DOI:** 10.1039/d4ra07408d

**Published:** 2024-12-17

**Authors:** Takahiro Suzuki, Fuka Nakamura, Kanon Ie, Masaaki Fujii, Masayuki Inoue

**Affiliations:** a Department of Chemistry, Faculty of Science, Tokyo University of Science 1-3 Kagurazaka Shinjyuku-ku Tokyo 162-8601 Japan staka@rs.tus.ac.jp; b Otsuma Ranzan Junior and Senior High School 558 Sugaya, Ranzan-machi Hiki-gun Saitama 355-0221 Japan; c Mathematics and Science Education, Graduate School of Science, Tokyo University of Science 1-3 Kagurazaka Shinjyuku-ku Tokyo 162-8601 Japan; d Research and Development Initiative, Chuo University 1-13-27 Kasuga Bunkyo-ku Tokyo 112-8551 Japan; e Laboratory for Chemistry and Life Science, Institute of Ingegrated Research, Institute of Science Tokyo 4259 Nagatsuta-cho, Midori-ku Yokohama 226-8503 Japan

## Abstract

A novel photochromic system was discovered, in which leucomethylene blue, reduced by l-ascorbic acid, was irradiated with 405 nm visible light, resulting in the solution undergoing a colorless-to-blue transition. The process was then repeated, with the solution returning to its colorless state when left undisturbed. This photochromic reaction is sensitive enough to be driven by a blue-violet LED lamp and enables figures to be drawn in solution with a laser pointer.

## Abstract 2

A novel photochromic system was discovered, in which leucomethylene blue, reduced by l-ascorbic acid, was irradiated with 405 nm visible light, resulting in the solution undergoing a colorless-to-blue transition.

## Graphical abstract

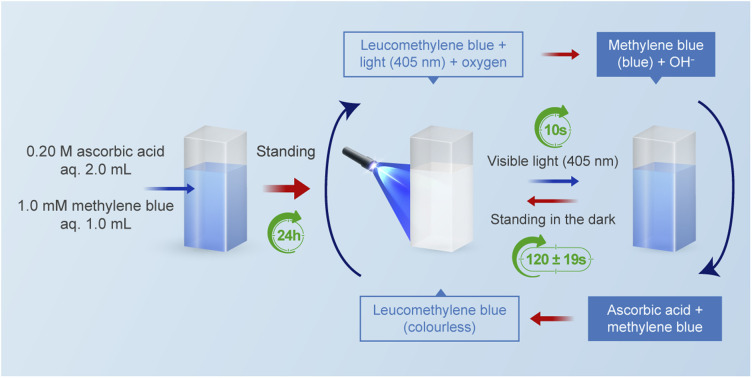

## 1. Introduction

Drawings can be made using pen on paper or lines in the sky using an airplane cloud. However, drawings are challenging to make in liquids. The ability to paint on a liquid safely and inexpensively can add value to the fields of culture and art, entertainment, education, medical care, and social welfare. Although pens and brushes are useless on liquids, light is advantageous, in that it can be transmitted and triggered remotely without directly contacting the liquid, in a similar manner to a paintbrush. Photochromic compounds such as spiropyrans dispersed in organic solvents or polymers reportedly become colored when irradiated with light.^1–4^ However, photochromic compounds are difficult to handle, and are challenging to produce in large quantities. In this study, we focused on methylene blue (Mb^+^), a water-soluble cationic dye that is inexpensive and produced on a large scale.^5–8^ Mb^+^ is known to accept hydrides either in a concerted or stepwise manner, depending on the reaction partner and conditions.^9,10^ The repetitive reaction in which an aqueous solution of Mb^+^ and a reducing agent turns blue when shaken and returns to a colorless state when set aside is referred as the “blue bottle experiment”.^11–20^ Additionally, leucomethylene blue (MbH) is reportedly colored by intense (100 W) near-ultraviolet (UV) light.^21–23^ We exposed the solution in the blue bottle experiment, using glucose as a reducing agent, to UV and visible light, but the color of the solution did not change. There are reports of methylene blue reduced with l-ascorbic acid being colored by UV light, but UV light is difficult to handle and too intense (48 W) for our purpose of drawing a picture in a liquid.^21^ Visible light, while easier to handle, has only been used to bleach the blue color at 665 nm.^21^

Based on these reports, we set about developing a photoinduced “blue bottle experiment” using weak visible light to enable painting in a liquid.

## 2. Experimental

### 2.1 Preparation of the solution and irradiation with ultraviolet and visible light

l-Ascorbic acid (AsA) and Mb^+^ were procured from the Kanto Chemical Co., Inc. The reaction solution was prepared by mixing 2.0 mL of 0.20 mol L^−1^ AsA solution with 1.0 mL of 1.0 × 10^−3^ mol L^−1^ Mb^+^ solution, and the mixture was left undisturbed for 24 h. Solution color changes were observed after irradiation with blue-violet light (*λ* = 405 nm) for 10 s using a light-emitting diode (LED) lamp (500 mW) and a laser pointer (200 mW). This experiment was conducted with the reaction solution maintained at 25 °C. UV-visible absorption at 665 nm and the potential were measured when the solution was exposed to light at wavelengths of 245, 365, 405, 415, 450, and 470 nm.

### 2.2 l-Ascorbic acid concentration

The AsA-concentration-dependent reduction of Mb^+^ in the reaction solution was investigated using AsA solutions with concentrations of 0.050, 0.010, 0.10, 0.20, 0.30, and 0.40 mol L^−1^. The reaction solutions were maintained at 25 °C and exposed to blue-violet light (*λ* = 405 nm) for 10 s, after which absorbances were measured and changes calculated.

### 2.3 pH Effects

The pH-dependence of the Mb^+^ decolorization reaction was examined by adjusting the pH of the reaction solution to 1.0, 2.0, 3.0, or 4.0 using hydrochloric acid or sodium hydroxide solutions. Reaction solutions were maintained at 25 °C and exposed to blue-violet light (*λ* = 405 nm) for 10 s, after which absorbances were measured and changes calculated.

### 2.4 Reaction temperature

The temperature-dependence of the Mb^+^ decolorization reaction was investigated by prepared solutions and then maintaining them at 15, 20, 25, 30, or 40 °C. The solutions were exposed to blue-violet light (*λ* = 405 nm) for 10 s, after which absorbances were measured and changes calculated.

### 2.5 Reaction cycling

To confirm that coloration and bleaching occurs repeatedly, we examined absorbance changes during cycling by exposing the reaction solution to blue-violet light (*λ* = 405 nm) for 10 s followed by standing in the dark for 100 s.

### 2.6 Optimal irradiation time for drawing patterns on a solution with violet-blue laser light

The optimal laser-light exposure time used to draw images on a solution with a laser pointer (*λ* = 405 nm) was determined by measuring absorbance values after the solution had been exposed to laser light for 3–20 s.

## 3. Results and discussion

### 3.1 Preparation of the solution and irradiation with ultraviolet and visible light

**Fig. 1:**
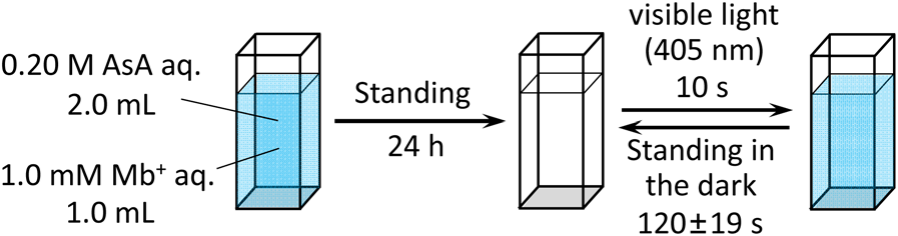
Overview of the experimental operation.

**Fig. 2:**
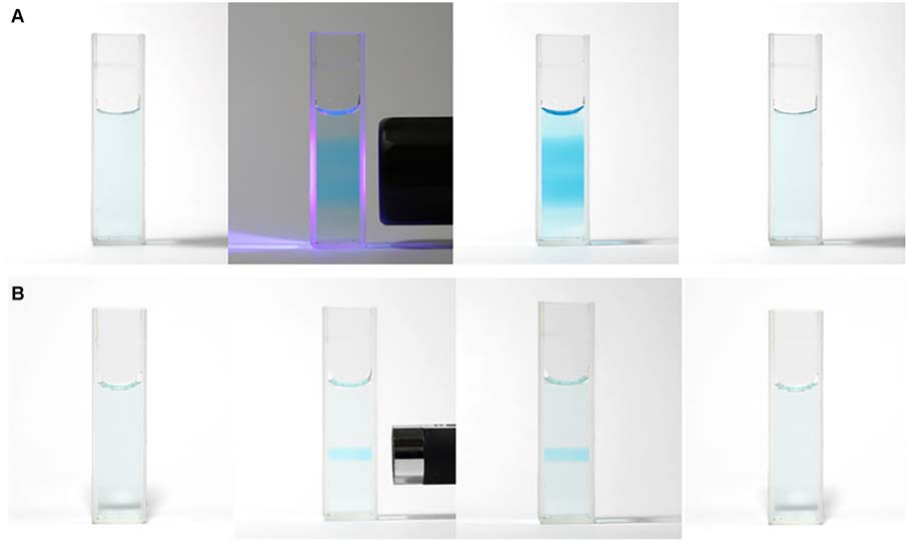
Changes in the color of the solution: (A) blue-violet LED lamp; (B) blue-violet laser pointer. Photographic images (from left to right) show solutions before irradiation, during irradiation, 5 s after the end of irradiation, and 120 s after the end of irradiation.

**Fig. 3:**
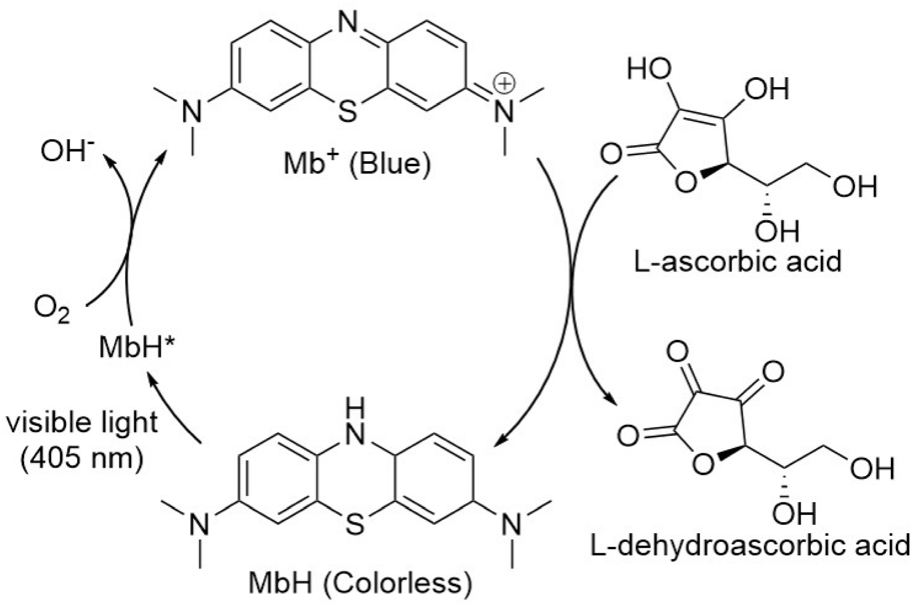
Reaction mechanism.

**Fig. 4:**
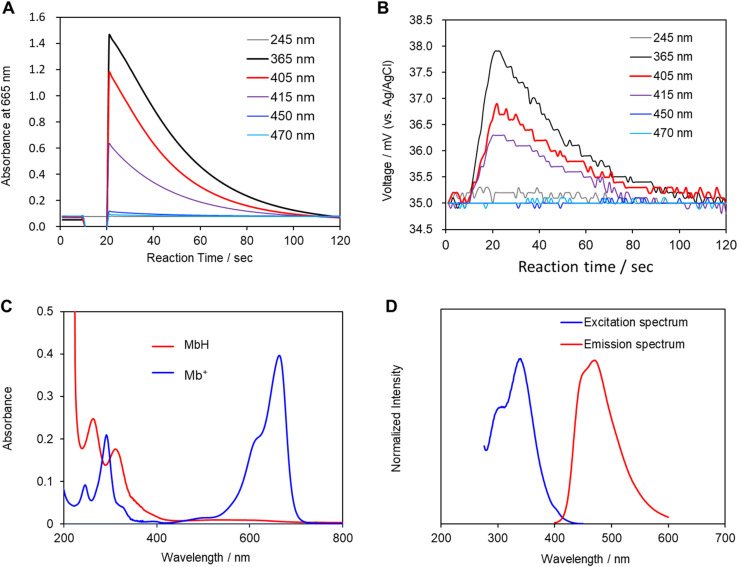
(A) Absorbances at a wavelength of 665 nm. (B) Change in electric potential when the solution was irradiated with light. Voltage values were obtained using a glassy carbon electrode as the working electrode, Pt wire as the counter electrode, and an Ag/AgCl electrode as the reference electrode. (C) UV-visible absorption spectra of MbH and Mb^+^. The MbH solution was prepared by adding sodium dithionite to an aqueous Mb^+^ solution. (D) Excitation spectrum of MbH at a fluorescence wavelength of 470.0 nm and fluorescence spectrum at an excitation wavelength of 340.0 nm.

Mb^+^ and AsA were mixed, resulting in Mb^+^ being reduced to colorless MbH. The irradiated area of the solution turned blue upon irradiation with blue-violet light (405 nm) for 10 s using an light-emitting diode (LED) lamp (500 mW) or a laser pointer (200 mW). The solution color gradually faded when irradiation was terminated, and was almost colorless after approximately 120 ± 19 s (Fig. 1 and 2, S1†). Error values were determined to 99% confidence based on 10 measurements using the Student's *t*-test. MbH absorbs light and enters an excited state when UV or blue laser light is introduced.^24^ Given that excited molecules are more reactive, they react immediately with oxygen to produce blue Mb^+^, which is subsequently reduced by AsA to colorless MbH;^20^ consequently the color fades over time. AsA has been reported to deprotonate Mb^+^ in the absence of a base and is the only reducing agent present under acidic conditions.^25^ We repeated this reaction (Fig. 3). The solution in the blue bottle experiment containing glucose and sodium hydroxide gradually oxidized MbH when it reacted with aerobic oxygen and turned blue as a consequence, even if the solution was set aside while exposed to air. However, the solution prepared in this study was acidic. Thus, MbH was more stable, and the solution did not turn blue when set aside when exposed to air; this solution became colored when irradiated with light. The UV-visible absorption at 665 nm and the potential were measured when the solution was irradiated with light at wavelengths of 245, 365, 405, 415, 450, and 470 nm (Fig. 4A and B). Higher absorbances were observed following irradiation at 365 nm (UV light), 405 nm, and 415 nm (visible light), which confirmed that MbH becomes colored (Fig. 4A). Sensitivity to ambient light was examined by exposing the solution to light from a fluorescent lamp and a white LED lamp; however, no coloring was observed (Fig. S2†). Regarding the measurement of electric potential, while the color of the solution was not influenced by the irradiated light during measurement, the potential value increased as a consequence, which is ascribable a change in the pH of the solution resulting from a reduction in the amount of MbH in response to light irradiation, as expressed by eqn (1) and (2) (Fig. 4B and S3†).1
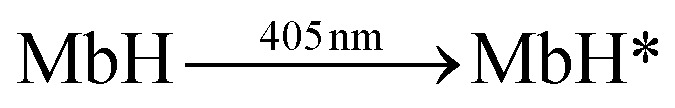

2 2MbH* + O_2_ → 2Mb^+^ + 2OH^−^

Changes in the solution due to irradiation with light were examined by measuring the pH and potential of the solution, in addition to its absorbance, because the concentration of hydrogen ions in the solution increases owing to the desorption of protons from MbH caused by irradiation with light. Moreover, Mb^+^ was investigated as a photosensitizer to produce singlet oxygen in photodynamic therapies such as cancer treatment.^26^ The photochemical reaction of Mb^+^ is due to the formation of an excited triplet state with a high quantum yield. However, in contrast to Mb^+^, the photochemistry of MbH has not been investigated extensively,^21^ which is expected given that MbH is colorless and absorbs light weakly in the near-UV region. When the UV-visible absorption spectrum of MbH was acquired, the absorption at approximately 405 nm was smaller than that in the UV region (Fig. 4C). Furthermore, weak absorption near 405 nm was observed when the excitation spectrum was acquired (Fig. 4D). LMB has been reported to typically absorb only UV-wavelength light, with two bands at 314 and 256 nm.^27,28^ However, the experimental results in the current study reveal that MbH absorbs visible light, which we ascribe to two factors. Firstly, the absorption band is located on the longer wavelength side, and the region near the original Franck-Condon-factor-broadened absorption band is directly excited by visible light. While the vibrational absorption spectra and electronic properties of Mb^+^ have previously been reported based on TD-DFT calculations,^29^ we are currently advancing research on this topic because insufficient research on the absorption band of MbH appears to exist. Secondly, two electronic states exist, with ultraviolet light traditionally used to excite electrons to higher energy or vibrational levels, with some of the energy dissipating through internal conversion to the lowest excited state. However, in this study, we believe that visible light directly excites electrons to the lowest excited state.

In any case, we experimentally demonstrated a phenomenon in which MbH absorbs visible light and undergoes coloration.

### 3.2 l-Ascorbic acid concentration

**Fig. 5:**
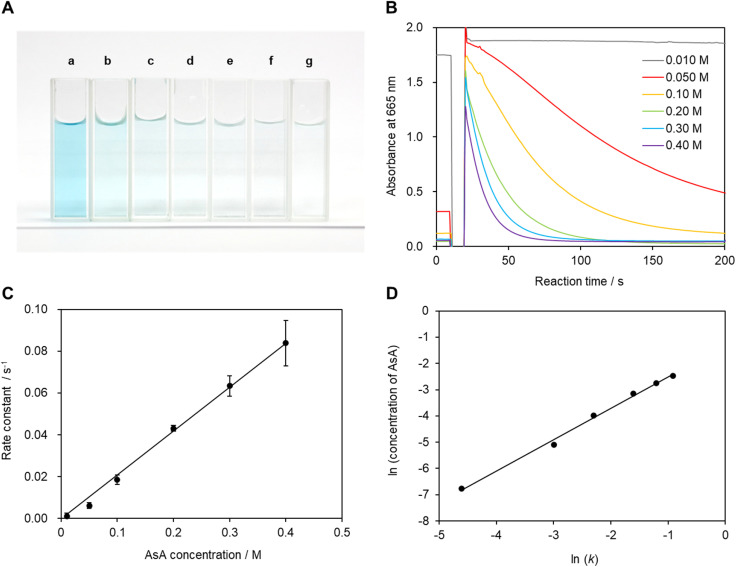
(A) Photographic images of the reaction solution before irradiation with light. (a) 0.010, b: 0.050, (c) 0.10, (d) 0.20, (e) 0.30, (f) 0.40, and (g) 0.50 mol L^−1^. (B) Absorbances at 665 nm measured 10 times. The displayed values correspond to the solution with the median reaction rate constant. (C) Relationship between the AsA concentration in solution and the reaction rate constant. Each point corresponds to the average of 10 measurements, with error bars shown at the 99% confidence level as determined by the Student's *t*-test. (D) Plot of ln(*k*) *vs.* log(AsA concentration).

The recorded absorbance indicates that the reaction solution is not completely transparent and appears to be slightly blue at low AsA concentrations. In particular, coloring and bleaching are barely observable to the naked eye at an ascorbic acid concentration of 0.01 mol L^−1^ (Fig. 5A and B). The time required for the reaction solution to decolorize increases with decreasing AsA concentration. The reaction mechanism is assumed to follow eqn (3). Eqn (4) can be expressed as a first-order reaction assuming that *k*_a_ is much larger than *k*_b_, which enables the rate constant to be determined.^30^3
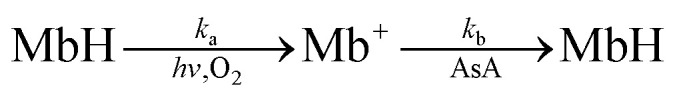
4
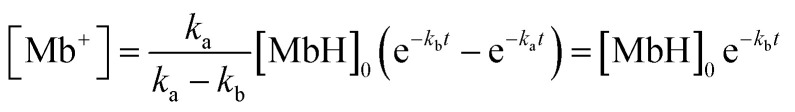


Fig. 5C shows the reaction rate constant as a function of AsA concentration when Mb^+^ is generated by irradiation with light. The reaction rate constant is expected to be proportional to AsA concentration if reduction is the primary reaction.^10,30,31^ Hence, these data provide evidence that AsA is involved in the reduction of Mb^+^. Therefore, we constructed a plot of log(*k*) as a function of log(AsA concentration) based on the time-dependent results acquired within the AsA concentration range (Fig. 5D), which reveals a slope of −0.029; hence the reaction order with respect to AsA concentration in this system is quite small. In other words, the reaction rate constant deviates slightly from the proportional relationship at high AsA concentrations, which is possibly ascribable to interactions between AsA molecules.

### 3.3 pH Effects

**Fig. 6:**
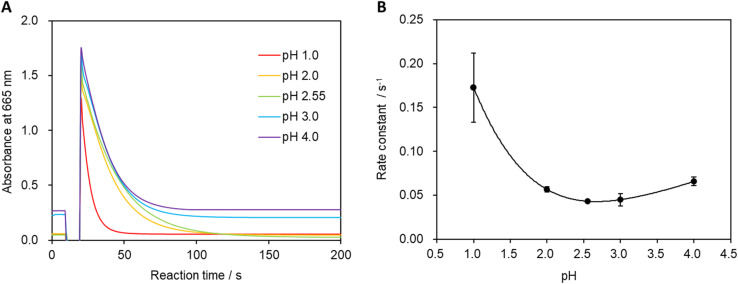
(A) Absorbance at a wavelength of 665 nm. Absorbance measurements were performed 10 times, and the displayed values correspond to the solution that exhibited the median reaction rate constant. (B) Relationship between solution pH and reaction rate constant. Measurements were conducted 10 times, and error bars correspond to 99% confidence as determined using the Student's *t*-test.

The pH of the system was varied, and absorbances were measured to calculate the reaction rate constant (Fig. 6A and B). Fig. 6B shows that the minimum reaction rate constant was recorded at pH 2.55 (unadjusted stock solution). A more-pronounced decrease in absorbance was observed under strongly acidic conditions, leading to an increase in the reaction rate constant. Additionally, the reaction rate constant gradually increased as the pH was increased beyond 2.55, which is believed to be due to the higher concentration of hydrogen ions in the solution under strongly acidic conditions that facilitate the formation of MbH from Mb^+^.^30^ Furthermore, the oxidation reaction of MbH becomes more favorable with increasing pH, which also contributes to the increase in the reaction rate constant.^32^

### 3.4 Reaction temperature

**Fig. 7:**
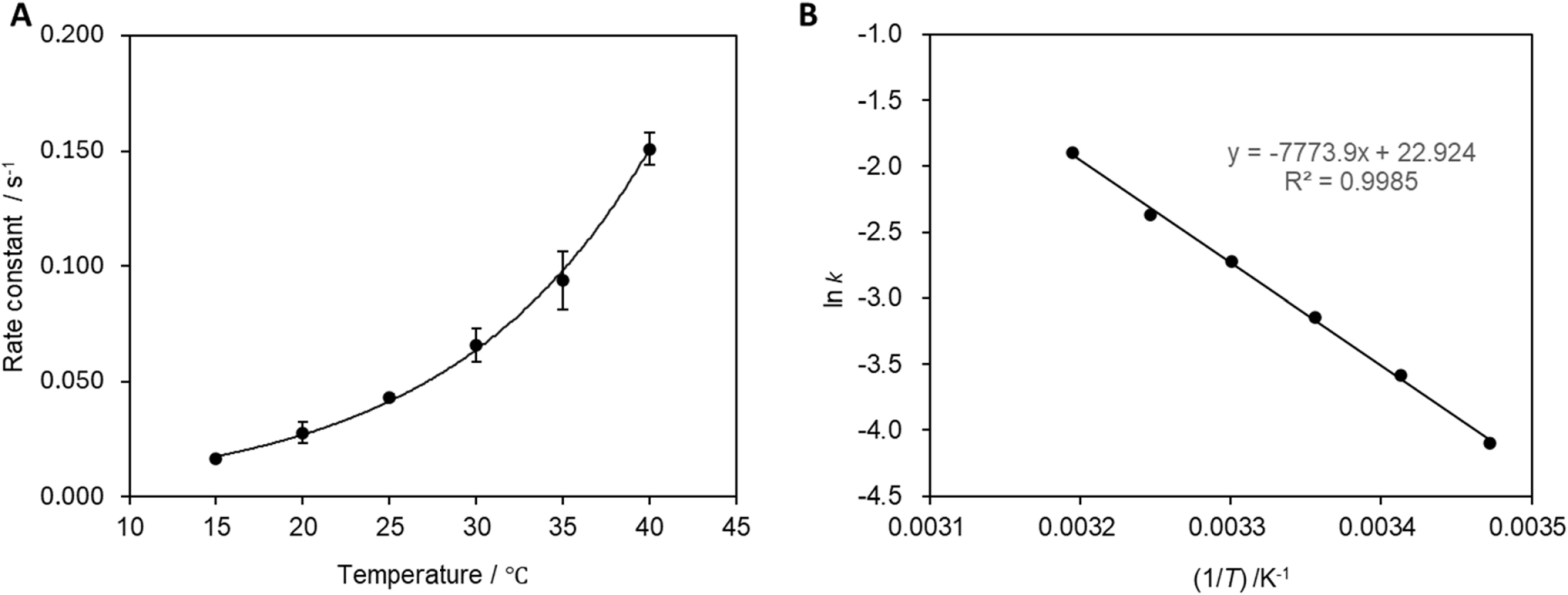
(A) Relationship between solution temperature the reaction rate constant. Measurements were performed 10 times, and error bars correspond to 99% confidence as determined using the Student's *t*-test. (B) Relationship between the reciprocal of the absolute temperature of the solution and ln(*k*).

The reaction rate constant was calculated at each temperature (Fig. 7A), which revealed that the reaction rate increases with increasing reaction temperature. The activation energy was determined to be 9.4 × 10^2^ J mol^−1^ from the slope of the relationship presented in Fig. 7B.

### 3.5 Repeated reaction cycling

**Fig. 8:**
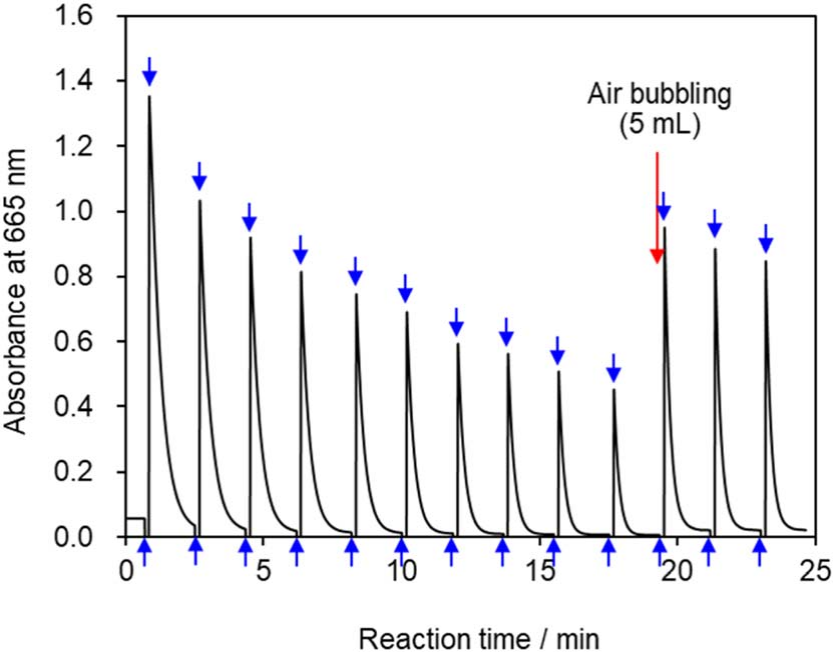
Absorbance *vs.* time profile at 665 nm. Upward arrows indicate irradiation of the reaction solution with light; downward arrows indicate cessation of irradiation.

Multiple cycles of solution coloration and decolorization confirmed that the solution is photochromic (Fig. 8). The reaction solution was placed in a cell for absorbance measurements, irradiated with blue-violet light (405 nm) for 10 s, and then set aside in the dark for 100 s. The UV-visible absorbance was measure following this operation, which was repeated multiple times. The absorbance value decreased gradually with repeated exposure to light; however, the absorbance value recovered when 5 mL of air was injected into the reaction solution *via* syringe followed by exposure to light which suggests that blue-violet light and oxygen are involved in the reaction, thereby providing evidence for the reaction pathway shown in Fig. 3. The solution can undergo more than 100 coloring and bleaching cycles as long as dissolved oxygen is not depleted. This reaction can be regarded as corresponding to the Mb^+^-catalyzed oxidation of AsA. The concentration of AsA decreases as the reaction is repeated, resulting in a longer time required to decolorize the solution. The repeatability of the reaction eventually ceases.

### 3.6 Optimal irradiation time for drawing patterns on a solution with violet-blue laser light

**Fig. 9:**
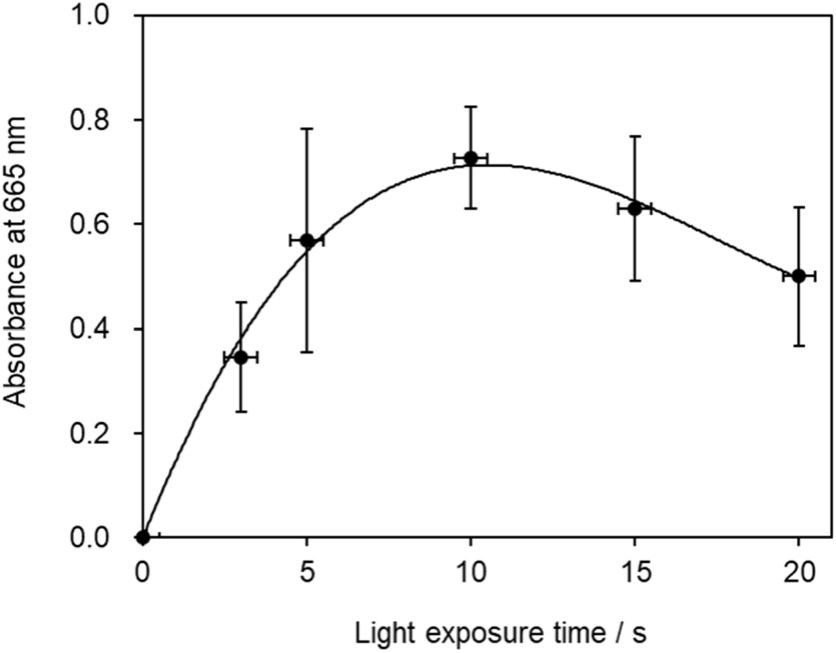
Absorbance *vs.* time profile at 665 nm.

The highest absorbance value was recorded when the solution was irradiated with laser light for 10 s, which also corresponded to the longest duration for which Mb^+^ remained in solution (Fig. 9). While a longer laser irradiation time is desirable, given that more Mb^+^ is generated in this mechanism, a clearly different result was observed, as the optimal irradiation time was determined to be 10 s, with longer irradiation resulting in a more-temporary blue-color state. This result is attributable to multiple factors. The first involves Mb^+^-photorecovery saturation. Considering that a longer irradiation time is superior assumes that the amount of Mb^+^ formed is proportional to the irradiation time. However, the amount of Mb^+^ formed is not proportional to the irradiation time if multiple MbH molecules are converted into Mb^+^ in the laser-light path, which then determines the optimal irradiation time. Secondly, a tradeoff relationship exists between the light-promoted formation of Mb^+^ and the reduction of Mb^+^ by AsA during irradiation with light. In addition, blue laser light contributes to bleaching of the blue color. The Mb^+^ absorption spectrum reveals weak absorption under blue light, and a fluorescent lamp has been reported to bleach the blue color of Mb^+^.^18^ Therefore, additional photochemistry may prevent an increase in the blue color. In addition, to investigate the effect of diffusion, we conducted an experiment in which the solution was absorbed by filter paper and irradiated with blue-violet light, which revealed that the diffusion rate was lower when the solution was absorbed by the filter paper than when it was placed in a Petri dish. Time was required for the color of the solution to fade even when on the filter paper. Therefore, the disappearance of the solution color is ascribable to diffusion and the reduction of Mb^+^ by AsA.

**Fig. 10:**
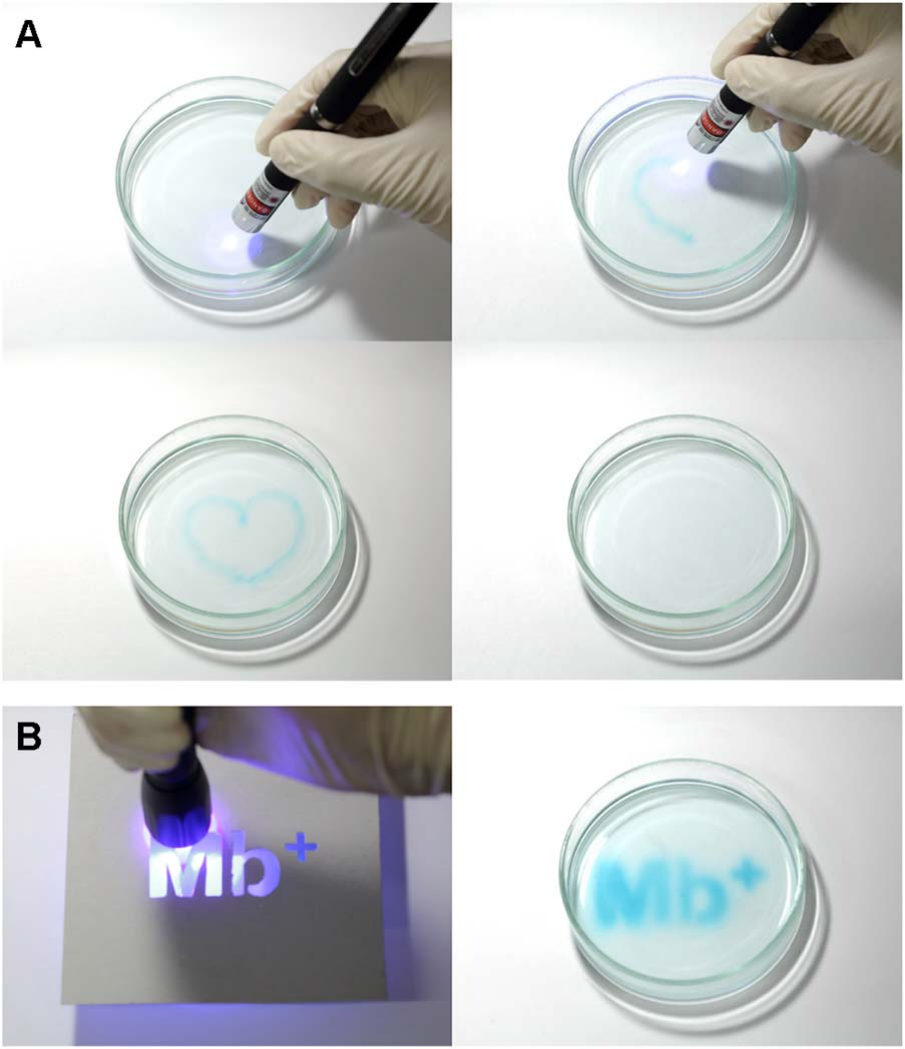
Solution being painted and returning to a colorless state after approximately 90 s. (A) Blue-violet laser pointer; (B) blue-violet LED lamp.

A 0.20 mol L^−1^ AsA solution and 1.0 × 10^−3^ mol L^−1^ Mb^+^ solution were mixed at a volume ratio of 2 : 1 for 24 h, and the mixture was placed in a Petri dish. A blue-violet laser pointer (405 nm) was used to draw pictures on the solution, which faded in approximately 90 s (Fig. 10A). In a similar manner, a blue-violet light can be used to draw pictures on the solution. In addition, only the areas exposed to the light became blue when the Petri dish was covered with a mask, as shown in Fig. 10B.

## 4. Conclusion

A solution of Mb^+^ and AsA was prepared and set aside to facilitate transition to a colorless state, after which it was irradiated with weak blue-violet light at 405 nm. The resulting activated MbH then reacted with oxygen to produce blue Mb^+^. This paper proposes a novel photochromism system that facilitates repeatedly observing the reaction in which the solution returns to a colorless state because Mb^+^ is reduced by AsA to form MbH when the solution is set aside. Although reactions such as the “blue bottle experiment” with Mb^+^ are well-known, using a visible light source enables novel applications that involve painting on solutions.

## Data availability

Data for this article, including the experimental details, are available in the ESI† of this paper.

## Author contributions

T. S., F. N. – conceptualization; T. S., F. N.,K. I. – data curation; T. S., F. N.,K. I. – formal analysis; T. S., M. I. – funding acquisition; T. S., F. N., K. I. – investigation; T. S., M. F. – methodology; T. S. – project administration; T. S., M. F., M. I. – resources; M. F. – software; T. S., M. I. – supervision; F. N. – validation; T. S., F. N. – visualization; T. S., F. N.,K. I. – writing – original draft; M. F., M. I. – writing – review & editing.

## Conflicts of interest

There are no conflicts to declare.

## Supplementary Material

RA-014-D4RA07408D-s001
