# Supplementary material for: Novel photochromic system using methylene blue reduction with l-ascorbic acid
Source: RSC Adv. 2024 Dec 17;14(53):39708–14. doi: 10.1039/d4ra07408d (PMC11651055; doi:10.1039/d4ra07408d)
Supplement: RA-014-D4RA07408D-s001 [file RA-014-D4RA07408D-s001.pdf]

Supplementary Information (SI) for RSC Advances.
This journal is © The Royal Society of Chemistry 2024

Supporting Information

Novel Photochromic System Using Methylene Blue Reduction with Ascorbic Acid

Takahiro Suzuki,*[a] Fuka Nakamura,[b] Kanon Ie,[c] Masaaki Fujii [d] and Masayuki Inoue [a]

a. Department of Chemistry, Faculty of Science, Tokyo University of Science, 1-3 Kagurazaka, Shinjyuku-ku, Tokyo 162-8601, Japan. E-mail: staka@rs.tus.ac.jp

b. Otsuma Ranzan Junior and Senior High School, 558 Sugaya, Ranzan-machi, Hiki-gun, Saitama 355-0221, Japan

c. Mathematics and Science Education, Graduate School of Science, Tokyo University of Science, 1-3 Kagurazaka, Shinjyuku-ku, Tokyo 162-8601, Japan.

d. Research and Development Initiative, Chuo University, 1-13-27 Kasuga, Bunkyo-ku, Tokyo 112-8551, Japan

S1. Effect of Light Intensity on Color

A 600-lux blue-violet LED light was used in this experiment. A lower maximum absorbance was recorded when the intensity of the light was reduced by a factor of ten (i.e., to 60 lux).

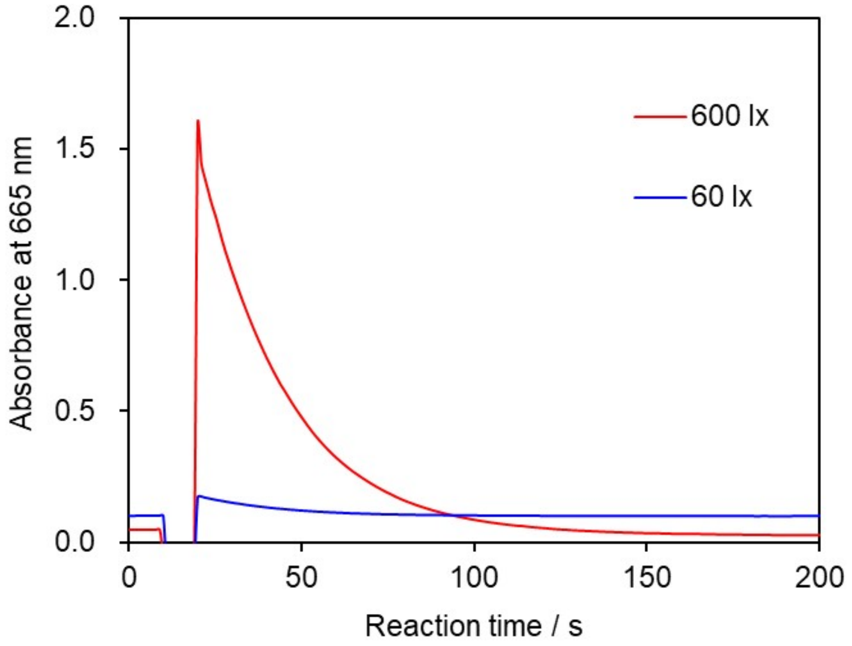

Fig. S1□Time-dependent changes in absorbance when the reaction solution was irradiated at different illuminances**.**

S2. Sensitivity of the Reaction Solution to Ambient Light (Light from a Fluorescent and a White LED Lamp)

Absorbances were measured when the reaction solution was exposed to light from a fluorescent lamp and a white LED lamp at a distance of 5 cm from the liquid surface. No solution coloring was observed.

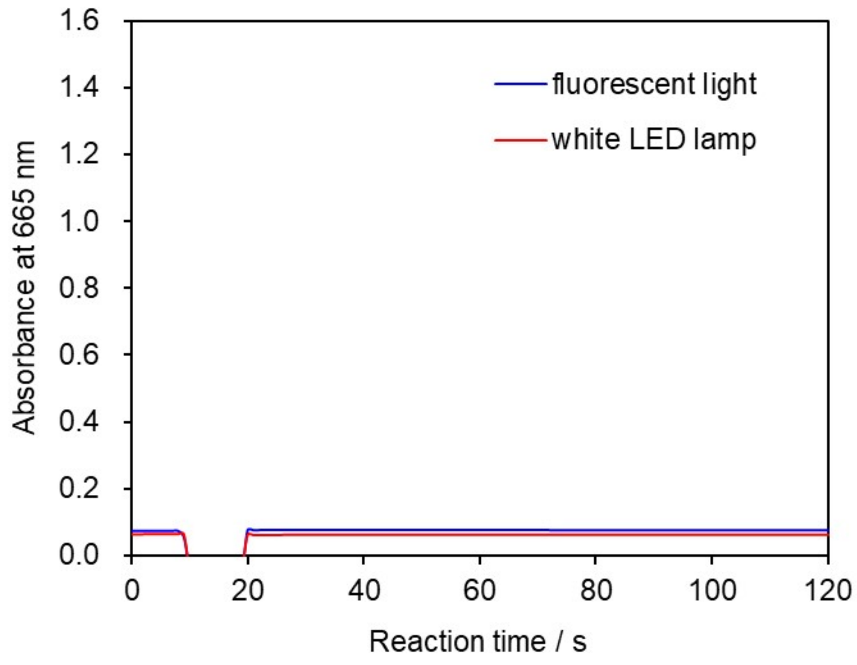

Fig. S2 Time-dependent changes in absorbance when exposed to fluorescent light and white light from an LED lamp.

S3. Reaction of solutions in blue bottle experiment with oxygen and blue-violet light

The solution for the blue bottle experiment was prepared by mixing 5.0 g of sodium hydroxide, 5.0 g of glucose, and 0.01 g of methylene blue in 250 mL of water according to Campbell's method, which was reported in 1963. The solution was colorless in a static state. When the solution was shaken or exposed to air or oxygen, the leucomethylene blue became oxidized, resulting in a blue color. However, irradiation with blue-violet light (405 nm) or ultraviolet light (365 nm) in the leucomethylene blue state did not color the solution.

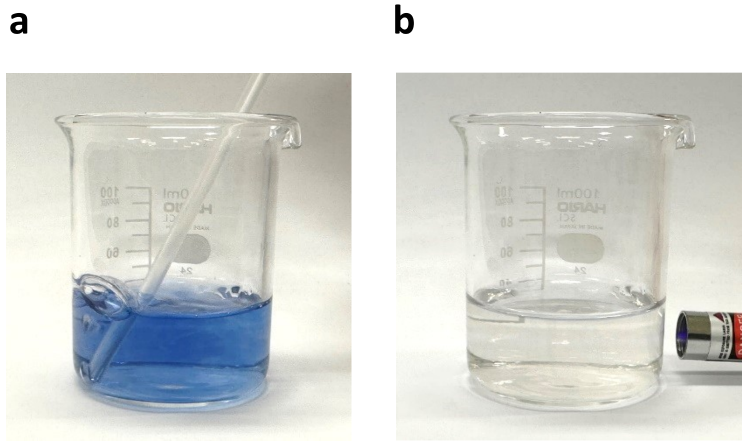

Fig. S3 Solution change in the blue bottle experiment. (a) Oxygen injection and (b) irradiation with blue-violet light (405 nm).
